# Multimorbidity and its social determinants among older people in southern provinces, Vietnam

**DOI:** 10.1186/s12939-015-0177-8

**Published:** 2015-05-30

**Authors:** Ninh Thi Ha, Ninh Hoang Le, Vishnu Khanal, Rachael Moorin

**Affiliations:** Institute of Public Health, 159 Hung Phu Street, District 8, Ho Chi Minh City, Vietnam; School of Public Health, Curtin University, Bentley, Western Australia Australia

**Keywords:** Developing country, Multimorbidity, Older people, Social determinants

## Abstract

**Background:**

Developing countries are poorly equipped for health issues related to ageing populations making multimorbidity challenging. As in Vietnam the focus tends to be on single conditions. Hence little is known about burden of multimorbidity. This study aimed to examine the prevalence and the determinants of multimorbidity among older people in Southern Vietnam.

**Methods:**

A cross-sectional study was conducted in two provinces of Southern Vietnam with a sample of 2400 people aged 60 years and older. The presence of chronic disease was ascertained by medical examination done by physicians at commune health stations. Information on social and demographic factors was collected using structured questionnaire. Univariate and multivariable logistic regression analyses were used to examine the factors associated with multimorbidity.

**Results:**

Nearly 40 % of older people had multimorbidity. Currently not working, and healthcare utilisation were associated with higher prevalence of multimorbidity. Living in urban areas and being literate were associated with lower prevalence of multimorbidity.

**Conclusion:**

The study found a high burden of multimorbidity among illiterate, especially those living in rural areas. This highlights the need for targeted community based programs aimed at reducing the burden of chronic disease.

## Background

Vietnam has experienced a remarkable change in demographic structure in recent decades. Life expectancy at birth increased from 66 years in 1990 to 72.8 years in 2009 [1]. The proportion of older population was 6.7 % in 1979 to 9.0 % in 2009 and has been estimated to reach to 16.6 % in 2029 and 26.1 % in 2049 [1, 2]. As a consequence of ageing of the population, there is a need for better understanding of the health status of older Vietnamese particularly in term of burden of chronic diseases so that appropriate responses to future needs of healthcare services can be developed.

Chronic diseases have been shown to be major causes of morbidity and mortality, accounting for 66 % of the overall disease burden in men and 77 % in women [3, 4]. Chronic disease among older people has been considered in many studies in Vietnam but these studies have focused on quality of life and daily care aspects [5, 6]. To our knowledge, the only one study investigating the prevalence of multimorbidity in Vietnam among adults aged 25–64 years old found a prevalence of 5.1 % among men and 9.3 % among women [7], however this study did not report prevalence in the elderly. Given the finding that the population of Vietnam is moving towards ageing, the prevalence of multiple chronic conditions is expected to increase and will tend to predominate in the older age groups. Since those with multimorbidity have a higher demand on healthcare services and increased healthcare costs [8], a better understanding of the burden of multimorbidity and its related factors among older people in Vietnam will provide evidence to estimate future demand for healthcare and aid in determining where health promotion programs should be targeted. This study aimed to examine the prevalence of multimorbidity as well as its associated factors among older people living in Southern provinces of Vietnam.

## Methods

### Settings and participants

The Southern Vietnam includes around 32.2 million population divided into two main areas namely the South East and the Mekong River Delta [9]. The proportion of population aged 60 years and older is approximately 10 % in both areas [9]. In 2010, the poverty rate was 2.3 % in the Southeast, in the Mekong River Delta it was six times higher (12.6 %), while the poverty rate of whole country was 10.7 % [9].

The health system in Vietnam consists of 4 levels: the national level with the Ministry of Health and central hospitals; the provincial level with the Provincial Department of Health and provincial hospitals; the district level with district health centres and district hospitals; and the communal level with commune health stations. Commune health stations provide primary healthcare services including preventive and treatment services and conducting national targeted health programs for people living in their catching areas. People receive diagnoses and routine care at the commune health station and are transferred to tertiary hospitals if needed [10].

### Study design and sampling

This population-based study was conducted among older people aged 60 years and older residents of Dong Nai in South East and Vinh Long in Mekong River Delta during September–October, 2010. Eligible participants were selected through multi-stage sampling. In the first stage, two districts in each province, one in urban and one in rural, were selected randomly. In the second stage, five communes were randomly selected from the list of communes in each district. From an updated list of household having people aged 60 years and older in each commune, 120 households were randomly selected for visiting. In each selected household, interviewers recruited randomly one older person for the study. To minimise the potential bias, all the older people living in the selected households, except visitors, were included in the list as being eligible for selection. Ninety-seven participants were absent at the time of conducting the survey and a further 26 were unwilling to participate. These potential participants were replaced with those from the nearest households having older people at same age and gender. The participation rate was 95 % which minimised participation bias in our study. A total sample of 2400 people aged 60 years and older was generated from the sampling strategy. Eligible participants were invited for interview and clinical examination in the commune health stations.

Ethical approval for the study protocol was obtained from the Institutional Review Board of Institute of Hygiene and Public Health, Ho Chi Minh City, Vietnam. All the participants of the study were provided with an explanation about the purpose of the study to avoid confusion with routine care they may have in their commune health stations. Participants were also informed about their right to not participate or withdraw from the study any time. If any inability to participate in this study that would not disadvantage them in their treatment and care at the commune health station. A written informed consent was obtained from all participants before conducting interviews.

### Instruments and data collection

The questionnaire included social demographic, and health related information including disease/morbidities the participants reported. The data collection was conducted by face-to-face interview using a structured questionnaire. The information about social demographic characteristics of the participants was gained via interviews at commune health stations by trained health staff familiar with the type of data they were collecting. The presence of chronic disease was determined by medical examination and chart review by general practitioners (GP). This was augmented (but not replaced) by an audit of prescriptions where participants self-reported a previous diagnosis of chronic disease. Thus, only those having medically documented chronic disease or currently receiving pertinent prescribed medications were classified as having chronic disease. All the participants were provided travel costs and services offered by the study were free of charge. All the activities for data collection were organised separately from a routine care work. Both interviewers and general practitioners were provided the training in interviewing skills, purpose of the study and their roles in the study before conducting data collection.

### Dependent variables

Information was collected about the following six broad groups of conditions: cardiovascular (including hypertension), digestive system (including liver), respiratory (including chronic obstructive pulmonary disease and tuberculosis), arthritis (including osteoarthritis), genitourinary, and diabetes. Individuals identified as having at least two of the conditions of interest were considered as living with multimorbidity [11].

### Independent variables

A number of factors were included as independent variables such as age, sex, marital status, education, and place of residence [5]. All participants were categorised into three age groups: 60–69 years, 70–79 years, and 80+ years. Marital status was grouped into two groups: married/cohabiting, and single/divorced/separated/widowed. Literacy was based on self-report and categorised as illiterate and literate. Place of residence was divided into urban and rural in accordance to the local administrative classification. Working status was categorised into currently working (still working in fields, or self-business or others for earning) and not working basing on the self-reporting of participants. Health behaviours were smoking status (never smoked and ever smoke) and alcohol intake (never alcohol and ever alcohol). Basic activities for daily living (ADLs) were assessed through the question “do you need any help for: (i) going to toilet (ii) getting dressed (iii) washing yourself and (iv) eating? Responses were categorised as need assistance for ADLs if the participants answered yes to any one of the options [12]. Weight and height were measured by health staff at the commune health stations to calculate body mass index. This was subsequently categorised into three groups according to the WHO recommendation for the Asian population: <18.5 kg/m^2^ underweight; 18.5–23 kg/m^2^ normal; > 23 kg/m^2^ overweight/obesity [13]. Healthcare utilisation was defined as the number of contacts with general practice, the number of medication prescriptions, and the number of referrals, categorised as yes if the participants reported using the healthcare services at least once over last week [14].

### Statistical analysis

Post-stratification weights were used in all analysis to adjust for under sampling in gender and place of residence in accordance with respective strata from national demographic data [9]. Data were presented with mean and standard deviation (SD) for continuous variables and with proportions for categorical variables. Univariate logistic regression models were used to examine the association between potential factors and the presence of multimorbidity (dichotomized “one chronic condition or none” vs. “at least two chronic conditions”) [11]. Variables that were statistically significant (*p*-value < 0.05) were further examined in multivariable logistic regressions through backward elimination. All variables were examined for interaction and multicollinearity before entering the logistic regression model. All analyses were performed using STATA 13.0.

## Results

The study included 2400 participants; the characteristics and prevalence of multimorbidity of the population studied are presented in Table 1. The mean age of participants was 72.6 years (SD 8.3 years). The majority was literate, 55.3 % married, and 70.6 % not working. Alcohol intake and smoking were not common (less than 20 % of participants) and only 6.5 % of older people reported that they needed assistance for basic activities for daily living. Underweight and overweight accounted for more than a half of the sample and nearly 30 % of respondents reported using healthcare service at least once in the last week. Chronic disease was present in 76.7 % of the sample with 37.7 % having only one condition and 39.2 % classified as living with multimorbidity.

**Table 1:** Demographic and socioeconomic characteristics of the study population

Characteristics	Sample		Weighted percentage
	*N*	%	% (95%CI)
Mean age in years (SD) 72.6 (8.3)			
Age group (years)			
60-69	913	38.0	36.9 (34.9-39.1)
70-79	936	39.0	38.7 (36.6-40.8)
80+	551	23.0	24.4 (22.5-26.3)
Gender			
Males	834	34.8	41.8 (41.7-41.7)
Females	1566	65.2	58.2 (58.2-58.2)
Marital status			
Married/cohabiting	1326	55.3	58.3 (56.3-60.2)
Single/ Divorced/ Widowed	1074	44.7	41.7 (39.8-43.7)
Literacy			
Illiterate	466	19.4	20.6 (19.0-22.3)
Literate	1934	80.6	79.4 (77.6-80.9)
Working status			
Current working	705	29.4	34.5 (32.4-36.5)
Not working	1695	70.6	65.5 (63.4-67.6)
Place of residence			
Rural	1200	50.0	69.9 (69.9-69.9)
Urban	1200	50.0	30.1 (30.1-30.1)
Alcohol intake			
Never alcohol	1952	81.7	77.0 (75.4-78.7)
Ever alcohol	435	18.2	23.0 (21.3-24.6)
Smoking			
Never smoked	1930	80.5	77.1 (75.3-78.2)
Ever smoked	467	19.5	22.9 (21.2-24.6)
BMI (kg/m^2^)			
Underweight (<18.5)	584	24.3	25.9 (24.1-27.8)
Normal weight (18.5 to 23)	1148	47.8	48.8 (46.7-51.0)
Over weight/obesity (> = 23)	668	27.8	25.3 (23.5-27.1)
Basic activities for daily living			
No need of assistance	1713	93.5	93.3 (92.1-94.3)
Needs assistance	155	6.5	6.7 (5.7-7.9)
Healthcare utilisation			
No	1698	70.7	71.2 (69.2-73.2)
Yes	702	29.3	28.8 (26.8-30.8)
Presence of chronic disease			
None	559	23.3	20.7 (19.1-22.5)
One chronic disease	900	37.5	37.7 (35.6-39.8)
Multimorbidity	941	39.2	41.6 (39.5-43.8)

Univariate logistic models showed that the prevalence of multimorbidity was significantly different across age groups, gender, marital status, education, working status, place of residence, need assistance for basis activities, and using healthcare services (Table 2). The prevalence of multimorbidity was significantly more common in the older age groups than in the younger age group, 45.9 % among 80 years or older and 43.4 % among 70–79 years old vs. 36.9 % among 60–69 years old (*p* = 0.03). It was also significantly more frequently observed among females than males, 43.8 vs. 38.6 % (*p* = 0.02), respectively. Prevalence of multimorbidity was significantly lower among literate participants than their counterparts (39 vs. 51.7 %, *p* < 0.001), significantly higher among those not working compared with currently working (43.6 vs. 37.9 %, respectively), lower among those living in urban than in rural (31.6 vs. 45.9 %, respectively), and higher among those who needed assistance for ADL compared with those who did not (51.5 vs. 40.9 %, respectively). Prevalence of multimorbidity was also positively associated with utilising healthcare services over the last week.

**Table 2:** Univariate associations between independent variables and multimorbidity

Characteristics	Multimorbidity		
	%	OR (95%CI)	*p*
Age group (years)			
60-69	36.9	1	0.03
70-79	43.4	1.3 (1.1-1.6)	
80+	45.9	1.4 (1.1-1.8)	
Gender			
Males	38.6	1	
Females	43.8	1.2 (1.03-1.5)	0.02
Marital status			
Married/cohabiting	39.9	1	
Single/ Divorced/ Widowed	44.0	1.2 (0.99-1.4)	0.06
Literacy			
Illiterate	51.7	1	
Literate	39.0	0.6 (0.5-0.7)	<0.001
Working status			
Current working	37.9	1	
Not working	43.6	1.3 (1.04-1.5)	0.01
Place of residence			
Rural	45.9	1	
Urban	31.6	0.5 (0.4-0.6)	<0.001
Alcohol intake			
Never alcohol	41.7	1	
Ever alcohol	41.1	0.9 (0.7-1.2)	0.82
Smoking			
Never smoked	41.5	1	
Ever smoked	41.8	1.01 (0.8-1.2)	0.91
BMI (kg/m^2^)			
Underweight (<18.5)	42.7	1	
Normal weight (18.5 to 23)	40.9	0.9 (0.7-1.2)	0.6
Over weight/obesity (> = 23)	42.3	1 (0.8-1.2)	0.9
Basic activities for daily activity			
No need of assistance	40.9	1	
Needs assistance	51.5	1.5 (1.1-2.2)	0.01
Healthcare utilisation			
No	39.3	1	
Yes	47.2	1.4 (1.1-1.6)	0.001

Results of the multivariate logistic regression analysis are presented in Table 3. While age, gender, and assistance for ADL had a crude association with multimorbidity these factors were not significant after adjusting for other variables in the model. In contrast, literacy, working status, place of residence and healthcare utilisation remained independently associated with multimorbidity. Being literate (OR 0.68 95%CI 0.54-0.85), and living in urban areas (OR 0.52; 95%CI 0.43–0.62) were independently associated with a lower likelihood of multimorbidity. Whereas, not currently working (OR 1.42 95%CI 1.16–1.74), healthcare utilization within the previous week (OR 1.39; 95 %CI 1.14–1.69) was independently associated with increased likelihood of multimorbidity.

**Table 3:** Backward multiple logistic regression analyses of significant factors associated with multimorbidity among older people

Characteristics	Multimorbidity		
	OR	95%CI	*p*
Literacy			
Literate vs. illiterate	0.68	0.54-0.85	0.001
Working status			
Not working vs. current working	1.42	1.16-1.74	0.001
Place of residence			
Urban vs. rural	0.52	0.43-0.62	<0.001
Healthcare utilisation			
Yes vs. No	1.39	1.14-1.69	0.001

## Discussion

This study is the first to provide an overview of the magnitude and socioeconomic correlates of multimorbidity among older adults in Vietnam. In ours study nearly 40 % of participants were ascertained to be living with multimorbidity. This result is in line with that of a study in Australia [15], but lower than that of a study in older people in rural Bangladesh [16]. It is also lower than that of a recent systematic review of studies mostly conducted in developed countries where the prevalence of multimorbidity among older people ranged from 55 %–98 % [8]. Such discrepancies may be due to the scope of the datasets; the differences in the age groups included in these studies, and methods used to assess the prevalence of disease [11, 17]. In addition, there is variation in the number and type of chronic diseases that are considered as multimorbidity by researchers. Regardless of these discrepancies in the definition of multimorbidity, our finding of higher prevalence than expected using existing literature in Vietnam [7] is important since multimorbidity is associated with disability, poor quality of life and higher need of health care utilisation, which put strain on healthcare systems [18]. Thus, a more context specific and up to date assessment of prevalence in Vietnam focused on older people in rural areas provides important information for planning of health services. In addition, since previous evidence suggests that chronic diseases are often diagnosed at critical or late stage in Vietnam as regular clinical or laboratory examination for early diagnosis is not a common practice [1], accurate estimates of prevalence obtained through studies such as ours are integral to the development of strategies to alleviate the considerable financial burden due to costly treatment for households caused by multimorbidity.

We found that the burden of multimorbidity varied according to socioeconomic factors. Women had significantly higher proportion of multimorbidity than men, however the difference was not significant when adjusted for other variables, indicating that other factors associated with multimorbidity are confounded by gender. This is an important distinction, since when evaluating independent factors associated with an outcome it is important to consider confounding. However, when determining which demographic group to focus on in the preventative or therapeutic space confounding is interpreted differently. Thus in our study the prevalence of multimorbidity being higher in women (regardless of why this is occurring, women per se or factors associated with women) has implications for the provision and burden on health services. The gender differential has been shown to exist in a previous study among adults in Vietnam [7] and other countries [8, 19]. Women tend to have longer life expectancy and worse health status compared with men, and women also differ from men in terms of social and biological factors [20]. This implies the need of more attention for women’s health and higher demand in healthcare for the population. Our study also agrees with previous studies showing that age is associated with multimorbidity [19, 21–23] and while confounded as per the discussion above this is still an important finding for planning of health services. Multimorbidity was significantly less common among literate people even adjusting for other variables showing that it is an independent factor and one that would be important to target in the preventative space. Studies conducted in Northern Vietnam [5, 7, 24] and other developing countries [16, 21] indicated that people with better education have less risk of chronic diseases. Education empowers an individual with knowledge and skill thus educated people can be more able to access health promotion information, and adopt healthy lifestyle [25]. Education can also shape job opportunities and earning potential, and influence living standards and healthcare [26].

Our study observed a significantly higher prevalence of multimorbidity in rural settings after adjusting for other variables. Other studies have shown the association between multimorbidity and place of residence, but with a lower prevalence in a rural area [20, 21]. Those studies conducted in a wide range of age groups that may explain the inverse association. This is a very important finding for our study as it shows that in the context of elderly Vietnamese, the rural populations are particularly at risk and pose a high and growing burden to the health care system. Healthcare utilisation was positively associated with multimorbidity. This finding was not unexpected and in line with other studies exploring the relationship between multimorbidity and utilisation of healthcare in South Africa [21], Catalonia [27], The Netherlands [14] and Europe [20]. The findings are likely to be due to the poorer general health of those living with multimorbidity; however there could also be some reverse causation bias whereby a high frequency of medical consults leads to more health conditions diagnosed. Regardless of the true nature of the relationship with the expected rise in ageing population in the coming decades, the result implied that more extensive health resources will be required [14].

Multimorbidity is common among older people and associated with healthcare utilisation that calls for a need of reviewing the organisation of healthcare services to meet the challenges of societies with a rapid increase in older population. Since developing countries are less prepared for health issues related to ageing population, more efforts should be allocated to educate primary healthcare workers and amend healthcare system in responding to the increase in multimorbidity, especially in rural areas. Our study indicates that in Vietnam intervention strategies and programs aiming to reduce burden of multimorbidity should be directed more to rural areas. Further research should look at mode of delivery of health services to older people in the community setting and feasibility of methods of community based health promotion programs to reduce the burden of chronic disease among the older population.

### Strengths and limitations

The findings of our study are important given the lack of evidence study that adds more evidence to the literature on the burden of multimorbidity in Vietnam. This is also the first study reporting multimorbidity burden among older people in the Southern provinces of Vietnam including larger community based samples. Importantly the findings of this study are generalisable to the older population in Vietnam due to the use of cluster sampling and adjustment for under sampling of gender and place of residence.

Given that the medical personnel determined the presence of morbidity, the study is more reliable than those using self-reported data. Our study was restricted to six groups of conditions. Since the more chronic conditions that are included the more patients with multimorbidity will be found, the prevalence of multimorbidity in our study may be underestimated. Nonetheless, the six condition groups included all important chronic morbidities and since the pattern of multimorbidity observed were similar to those observed in many other studies we are confident that our study captured the majority of important multimorbidity.

This was a cross-sectional study, and thus causal relationships between independent variables and the prevalence of multimorbidity cannot be determined, rather evidence on prevalence and factors associated with the likelihood of multimorbidity have been evaluated. A strength of the study was our ability to report findings for both the univariate and multivariate models has also provided important evidence regarding stratified burden (univariate results) and independent associations (multivariate results) that are useful to health system policy makers.

## Conclusion

The results of our study showed that multimorbidity are common among the older population of Southern Vietnam particularly in women and those of older age. Living in rural areas, being illiterate and not currently working were independently associated with multimorbidity. Those with multimorbidity were significantly more likely to have increased health service utilisation confirming the increased burden on health systems. The findings of our study indicate that intervention strategies and community based health promotion programs to reduce the burden of chronic disease among the older population should be directed more towards women and the elderly especially in rural areas.
